# Integral Role of the Mitochondrial Ribosome in Supporting Ovarian Function: MRPS7 Variants in Syndromic Premature Ovarian Insufficiency

**DOI:** 10.3390/genes13112113

**Published:** 2022-11-14

**Authors:** Brianna L. Kline, Sylvie Jaillard, Katrina M. Bell, Shabnam Bakhshalizadeh, Gorjana Robevska, Jocelyn van den Bergen, Jérôme Dulon, Katie L. Ayers, John Christodoulou, Michel C. Tchan, Philippe Touraine, Andrew H. Sinclair, Elena J. Tucker

**Affiliations:** 1 Murdoch Children’s Research Institute, Royal Children’s Hospital, Melbourne, VIC 3052, Australia; 2 IRSET (Institut de Recherche en Santé, Environnement et Travail), INSERM/EHESP/Univ Rennes/CHU Rennes–UMR_S 1085, F-35000 Rennes, France; 3 CHU Rennes, Service de Cytogénétique et Biologie Cellulaire, F-35033 Rennes, France; 4 Department of Paediatrics, University of Melbourne, Melbourne, VIC 3052, Australia; 5 Department of Endocrinology and Reproductive Medicine, AP-HP, Sorbonne University Medicine, Centre de Référence des Maladies Endocriniennes Rares de la Croissance et du Développement, Centre des Pathologies Gynécologiques Rares, 75231 Paris, France; 6 Department of Genetic Medicine, Westmead Hospital, Sydney, NSW 2145, Australia

**Keywords:** premature ovarian insufficiency, Perrault syndrome, MRPS7, mitochondrial disease, ovarian dysfunction, mitochondrial ribosome

## Abstract

The mitochondrial ribosome is critical to mitochondrial protein synthesis. Defects in both the large and small subunits of the mitochondrial ribosome can cause human disease, including, but not limited to, cardiomyopathy, hypoglycaemia, neurological dysfunction, sensorineural hearing loss and premature ovarian insufficiency (POI). POI is a common cause of infertility, characterised by elevated follicle-stimulating hormone and amenorrhea in women under the age of 40. Here we describe a patient with POI, sensorineural hearing loss and Hashimoto’s disease. The co-occurrence of POI with sensorineural hearing loss indicates Perrault syndrome. Whole exome sequencing identified two compound heterozygous variants in *mitochondrial ribosomal protein 7* (*MRPS7*), c.373A>T/p.(Lys125*) and c.536G>A/p.(Arg179His). Both novel variants are predicted to be pathogenic via in-silico algorithms. Variants in *MRPS7* have been described only once in the literature and were identified in sisters, one of whom presented with congenital sensorineural hearing loss and POI, consistent with our patient phenotype. The other affected sister had a more severe disease course and died in early adolescence due to liver and renal failure before the reproductive phenotype was known. This second independent report validates that variants in *MRPS7* are a cause of syndromic POI/Perrault syndrome. We present this case and review the current evidence supporting the integral role of the mitochondrial ribosome in supporting ovarian function.

## 1. Introduction

Premature ovarian insufficiency (POI) is a common cause of infertility in women, characterised by elevated follicle-stimulating hormone (FSH) and menstruation disturbances under the age of 40 [1]. POI is believed to have a strong genetic basis, yet many POI patients have an undefined genetic cause [1]. Elucidating POI causative genes provides an opportunity for early interventions, treatments, minimises co-morbidities and maximises fertility potential.

Perrault syndrome (PS) is a rare autosomal recessive condition characterised by sensorineural hearing loss (SNHL) in both sexes in addition to POI in affected females [2]. Hearing loss varies from mild adult-onset SNHL to severe and congenital SNHL [2]. The severity of ovarian dysfunction can vary from secondary amenorrhea and POI to a complete failure of ovarian development (streak gonads), failed pubertal development and primary amenorrhea. Reproductive development and function are normal in PS patients with an XY karyotype. PS is genetically and clinically heterogenous and is challenging to diagnose as the connection between auditory and reproductive symptoms is frequently overlooked [3]. In addition to SNHL, diverse neurological features have also been observed in patients. This includes generalised developmental delay, motor/peripheral neuropathy and ataxia [2].

To date, variants in eleven different genes have been associated with Perrault syndrome. These genes are associated with mitochondrial protein maintenance (*CLPP*), mitochondrial rRNA chaperones (*ERAL1*), mitochondrial translation (*LARS2, HARS2, RMND1*), mitochondrial ribonuclease activity (*PRORP*), mtDNA maintenance (*TWNK, TFAM*), fatty acid oxidation/steroid metabolism (*HSD17B4*), peroxisome biogenesis (*PEX6*) and lipid synthesis (*GGPS1*) [3,4,5,6]. Variants in these eleven genes account for approximately 40% of PS cases. Therefore, the underlying genetic cause in ~60% of PS cases remains unknown, and novel PS genes likely await discovery [3].

The mitochondrial ribosome (mitoribosome) performs protein synthesis within the mitochondria of eukaryotic cells. Human mitoribosomes consist of two distinct subunits, the 39S large subunit (39S-LSU) and the 28S small subunit (28S-SSU). The 39S-LSU is comprised of 16S rRNA and approximately 48 associated proteins [7]. The 28S-SSU is comprised of 12S rRNA and approximately 30 proteins [7]. Together, these components consist of approximately 80 proteins, 36 of which are mitochondria-specific. Thus far, nine of these subunit proteins have been associated with human mitochondrial disorders [8].

Genes encoding subunits of the 39S-LSU that have been associated with human disease include *MRPL3* (OMIM: 607118), *MRPL12* (OMIM: 602375), *MRPL44* (OMIM: 611849) and *MRPL24* (OMIM: 611986) [8]. In two instances, *MRPL3* has been associated with autosomal recessive combined oxidative phosphorylation deficiency (COXPD) and cardiomyopathy [9]. There is also suggestive evidence of heterozygous *MRPL3* variants being associated with Tourette Syndrome/chronic tick disorders based on co-segregation in seven affected individuals in a single pedigree [10]. Variants in *MRPL12* have been associated with autosomal recessive growth retardation, neurological defects and oxidative phosphorylation (OXPHOS) deficiency [11]. Autosomal recessive *MRPL44* variants were observed in sisters who presented clinically with infantile cardiomyopathy [12]. Finally, homozygous missense variants within *MRPL24* (OMIM 611986) were observed in a child with cerebellar atrophy, combined deficiency of complexes I and IV and choreoathetosis [13] (Table 1).

With respect to the 28S-SSU, autosomal recessive variants in *MRPS2* (OMIM: 611971) have been found to cause recurrent hypoglycaemia and lactic acidosis [8,14]. A homozygous nonsense variant in *MRPS16* (OMIM: 609204) resulted in agenesis of the corpus callosum, dysmorphism and fatal neonatal lactic acidosis [15]. A child with a homozygous missense mutation in *MRPS23* (OMIM: 611985) presented with infantile hepatic disease [16]. Variants in *MRPS34* (OMIM: 611994) have been described as a cause of the severe neurological disorder Leigh syndrome [14,17]. Intrauterine growth retardation, developmental delay and craniofacial abnormalities were observed in a patient with a homozygous variant in *MRPS28* (OMIM: 611990) [18]. Homozygous variants in *MRPS14* in another patient (OMIM: 611978) resulted in hypertrophic cardiomyopathy, lactic acidosis, developmental delay and muscle hypotonia [19]. Variants in *MRPS39* (OMIM: 614918) have been described as a cause of Leigh syndrome [20]. A patient with cerebral palsy, partial corpus callosum agenesis and mitochondrial myopathy was found to have homozygous variants in *MRPS25* (OMIM: 611987) [21]. Variants in *MRPS22* (OMIM:605810) have been associated with several clinical phenotypes. These include Cornelia de Lange-like dysmorphic features, brain abnormalities, and hypertrophic cardiomyopathy [22]. Interestingly, pathogenic variants in *MRPS22* have also been shown to cause isolated POI inherited in an autosomal recessive manner [23] (Table 1).

**Table 1:** Mitochondrial ribosome subunits associated with disease.

Gene	OMIM	Inheritance	Clinical Presentation
**Large 39S-LSU**			
*MRPL3*	607118	AR/AD	COXPD and cardiomyopathy. Suggestive implication in Tourette’s syndrome/chronic tick disorder [9,10]
*MRPL12*	602375	AR	Growth retardation, neurological defects and OXPHOS deficiency [11]
*MRPL24*	611986	AR	Cerebellar atrophy, combined defect of complexes I and IV and choreoathetosis [13]
*MRPL44*	611849	AR	Infantile cardiomyopathy [12]
**Small 28S-SSU**			
*MRPS2*	611971	AR	Hypoglycaemia and lactic acidosis [8,14]
*MRPS7*	611974	AR	Sensorineural hearing loss, hepatic and renal failure delayed pubertal onset, primary hypogonadism [24].
*MRPS14*	611978	AR	Hypertrophic cardiomyopathy, lactic acidosis, developmental delay and muscle hypotonia [19]
*MRPS16*	609204	AR	Agenesis of corpus callosum, dysmorphism and fatal neonatal lactic acidosis [15]
*MRPS22*	605810	AR	Cornelia de Lange-like dysmorphic features, brain abnormalities, hypertrophic cardiomyopathy and isolated POI [22,23]
*MRPS23*	611985	AR	Hepatic disease [16]
*MRPS25*	611987	AR	Cerebral palsy, partial corpus callosum agenesis and mitochondrial myopathy [21]
*MRPS34*	611994	AR	Neurological dysfunction, Leigh syndrome [14,17]
*MRPS39*	614918	AR	Leigh syndrome [20]

AD = Autosomal dominant, COXPD = Combined oxidative phosphorylation deficiency, POI = Premature ovarian insufficiency, AR = Autosomal recessive, OXPHOS = Oxidative phosphorylation, LSU = large subunit, SSU = small subunit.

Patients with variants in *mitochondrial ribosome protein 7* (*MRPS7*) (OMIM:611974), also a 28S-SSU protein, have been reported in only one family, with affected sisters presenting with phenotypes of varying severity. Both had sensorineural hearing loss; one died in early adolescence due to hepatic and renal failure whereas the other had delayed pubertal onset and primary hypogonadism [24]. The paucity of *MRPS7* cases described in the literature limits conclusive genotype: phenotype association.

Here, we provide independent replication of bi-allelic *MRPS7* variants in association with syndromic POI. Novel compound heterozygous *MRPS7* variants were identified via whole exome sequencing (WES) in a patient with a phenotype concordant with, although milder, than that previously associated with the gene [24]. We provide a clinical update on the previously reported patient and compare her phenotype to the present case. This provides evidence to support causation, elevating *MRPS7* from a candidate gene to a diagnostic gene for mitochondrial disorders and syndromic POI. This work also highlights the important role of the mitochondrial ribosome in ovarian development and function.

## 2. Materials and Methods

### 2.1. Ethics

Written informed consent was obtained from all participants. All procedures were approved by the Human Research Ethics Committee of the Royal Children’s Hospital, Melbourne (HREC/22073).

### 2.2. Participants

Patients were recruited after clinical consultation as part of our ongoing research program investigating the genetics of POI. Family and personal medical histories were collated and are included in Table 2. The patient was diagnosed with POI at age 25. POI was defined by menstrual disturbance and elevated FSH (>20 mIU/mL) measured twice at least one month apart as per the European Society of Human Reproduction (ESHR) guidelines (https://www.eshre.eu/Guidelines-and-Legal/Guidelines/Management-of-premature-ovarian-insufficiency.aspx, accessed on 10 November 2022) [25]. There was no history of ovarian surgery, infection or gonadotoxic therapy that could explain her condition. Karyotyping/microarray was performed to confirm normal 46, XX chromosomal complement, and she was negative for *FMR1* premutation testing and ovarian auto-antibodies.

### 2.3. General Molecular Techniques

Genomic DNA was extracted from EDTA-blood manually with the NucleoSpin^®^ Blood XL kit (Macherey-Nagel) or with an automated system, Hamilton Microlab STAR and Nucleospin^®^ Blood L kit (Macherey-Nagel), and was assessed by NanoDrop^TM^ 1000 spectrophotometer and Qubit dsDNA BR Assay (Thermo Fisher Scientific). Selected variants were validated by Sanger sequencing using BigDye v3.1 Terminators (Applied Biosystems) and ABI 3130X. Long-range PCR was performed using the GoTaq^®^ Long PCR Master Mix Promega kit. Primer sequences are available on request.

### 2.4. Whole-Exome Sequencing (WES)

DNA underwent WES at the Victorian Clinical Genetics Service (VCGS), with exome capture using SureSelect Human All Exon V6 (Agilent) and sequencing performed on the NextSeq 500/550 (Illumina). WES data were processed using Cpipe [26] and deposited into *seqr* for analysis (https://seqr.broadinstitute.org/, accessed on 14 December 2021).

We performed two phases of analysis as previously described [1]: the first was gene-centric using a candidate POI gene list, and the second was variant-centric. Variant-centric analysis focused on high-priority variants (frameshift, nonsense or splice site variants) in any gene and with any inheritance, or potentially bi-allelic moderate-high priority variants (missense, in-frame indels, frameshift, nonsense or splice spite). Only variants with MAF < 0.005 in 1000 genomes and gnomAD (https://gnomad.broadinstitute.org/, accessed on 14 December 2021) and with high quality scores (Q > 50 and allele balance >25) were considered. Variant pathogenicity was predicted in silico using Mutation Taster (http://www.mutationtaster.org/, accessed on 14 December 2021), Polyphen-2 (http://genetics.bwh.harvard.edu/pph2/, accessed on 14 December 2021), SIFT/Provean (http://provean.jcvi.org/, accessed on 14 December 2021), and CADD (Combined Annotation-Dependent Depletion) (https://cadd.gs.washington.edu/snv, accessed on 14 December 2021). The conservation of affected residues was assessed by Multiz Alignments of 100 vertebrates (UCSC Genome Browser https://genome.ucsc.edu/, accessed on 14 December 2021).

### 2.5. Variant Phasing

For variant phasing, DNA from the corresponding patient was cloned using the pGEM-T Easy Vector System (Promega Corporation, Madison, WI, USA).

A large genomic region (3.7 kb) bearing the two variants was first amplified by PCR using GoTaq^®^ Long PCR Master Mix (Promega Corporation, Madison, WI, USA). PCR products were purified using ExoSAP-IT PCR Product Cleanup Reagent (Thermo Fisher Scientific, Waltham, MA, USA). The addition of an A-tail to the amplicon was performed using GoTaq^®^ Flexi DNA polymerase (Promega Corporation, Madison, WI, USA) and 0.2 mM dATP (0.2 mM of final concentration) in a thermal cycler at 70 °C for 20 min.

Ligation was then performed in pGEM-T Easy Vector (Promega Corporation) as per the manufacturer’s protocol. XL10-Gold Ultracompetent cells (Agilent Technologies, Santa Clara, CA, USA) were transformed as per the manufacturer’s protocol and were plated on ampicillin/X-Gal/IPTG (Isopropyl β-D-1 thiogalactopyranoside) plates overnight at 37 °C.

White colonies were selected for colony screening by PCR with nested primer pairs flanking the two patient variants. (Forward: 5’ATGGGAGTAAAGGGCAAGGT3’ and Reverse: 5’ATCCCACTGCACCAGCTAGA3’ to capture the exon 4 variant, c.373A>T, and Forward: 5’TGAGGGAAGCTCGAAGAAAA3’ and Reverse: 5’GCCCACCCTTACAAAGGAAC3’ to capture the exon 5 variant, c.536G>A).

Sanger sequencing was performed by the Australian Genome Research Facility (AGRF).

## 3. Results

### 3.1. Diagnosis of Syndromic Premature Ovarian Insufficiency

**Figure 1:**
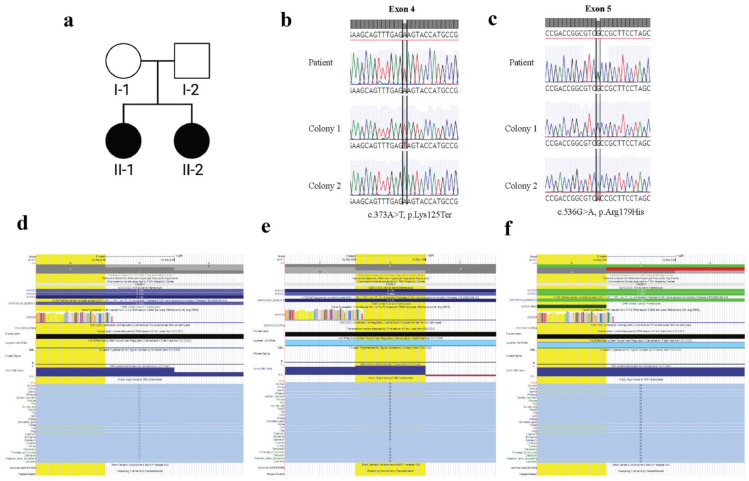
(**a**) Pedigree of the affected Proband (II-1) and sister (II-2) with two unaffected parents from a non-consanguineous union. (**b**) *MRPS7* variants are bi-allelic. Sanger sequencing of genomic DNA (patient) and individual colonies show the heterozygous exon 4 variant in the patient and its presence in Colony 1 but absence in Colony 2. (**c**) Sanger sequencing of genomic DNA (Patient) and individual colonies show the heterozygous exon 5 variant in the patient and its presence in Colony 2 but absence in Colony 1. (**d**) Patient residue p.Lys125* is evolutionarily conserved. (**e**) Patient residue p.Arg179His is evolutionary conserved. (**f**) Previously reported patient residue [24].

A 25-year-old woman was referred for evaluation after experiencing secondary amenorrhea. She underwent normal pubertal development and was of normal height and weight upon consultation. The family had no known consanguinity. The patient was diagnosed with sensorineural hearing loss (SNHL) at age 9 and has a clinical diagnosis of Hashimoto’s disease that is well-managed. No other autoimmune conditions are reported in the family. The proband has a similarly affected sister with congenital hearing loss and POI diagnosed at 21 years. Co-occurrence of SNHL and POI is indicative of Perrault syndrome in the sibling pair (Figure 1a).

Hormone evaluation of the proband revealed elevated FSH levels of 102 IU/mL (normal range 1.5–12.4 IU/mL), suggestive of POI. Estradiol was low at 29 pg/mL (normal range 30–400 pg/mL). Thyroid stimulating hormone (TSH) was elevated at 12.89 mIU/L (normal range 0.5 to 5.0 mIU/L). TSH levels remain consistent with hypothyroidism and the patient’s diagnosis of Hashimoto’s disease. The patient tested positive for anti-thyroid peroxidase antibodes at 102 IU/mL (reference < 16 IU/mL). FSH and oestradiol levels remain consistent with those of a post-menopausal woman.

An abdominal ultrasound was performed at 25 years of age. Scans revealed the presence of ovaries, with the right ovary measuring 18 mm in length and 4.5 mm in width and the left ovary measuring 14 mm in length and 6 mm in width (compared to an average width of 15–30 mm in controls). Two microfollicles were observed in the right ovary, and no follicles were evident in the left. Bone mineral density (BMD) scans did not indicate an osteoporotic phenotype.

### 3.2. Whole Exome Sequencing Identifies MRPS7 Variants

The proband underwent whole-exome sequencing (WES) to investigate the cause of syndromic POI. The median exonic depth of coverage was 93, with 98.7% of bases having a depth greater than 10. There were three moderate-high priority variants detected in POI candidate genes, however, there was minimal evidence to suggest these heterozygous variants could be causative. In contrast, variant-centric analysis focusing on moderate-high priority potentially bi-allelic variants flagged *MRPS7* as a gene of interest, as did variant-centric analysis focusing on high priority variants in any gene and with any mode of inheritance (Table 3).

The top candidate variants were two heterozygous variants in *MRPS7*. The first is a nonsense variant (c.373A>T), introducing a premature termination codon (p.Lys125*). This change is predicted to be disease-causing by in-silico algorithms, Table 4. This variant is in exon 4 of the 5 exons of *MRPS7*, meaning that the variant would likely cause nonsense-mediated decay and result in a loss of function (Lof) allele. * interested gene.

The second variant is a missense (c.536G>A), resulting in a substitution of arginine to histidine (p.(Arg179His)) in exon 5. In-silico algorithms consistently predict this variant to be damaging (Table 4 and Supplementary Table S1).

Both variants observed in *MRPS7* effect residues that are evolutionarily conserved (Figure 1d,e). p.Arg179 is conserved in mammals, reptiles and fish. This conservation reflects the likely integral role for this residue in the structure and/or function of MRPS7. The substitution is that of an arginine to a histidine at position 179 in MRPS7, and is predicted to be pathogenic. Histidine is significantly smaller in size compared to arginine. This size differential is predicted to impact the neighbouring hydrogen bond with glutamic acid at position 153 [27]. Further, the substitution from arginine to histidine alters the charge from positive to neutral and is predicted to disrupt salt bridges with Glu153 and Asp176. We therefore predict that the patient variant will disrupt the overall stability of the MRPS7 protein. Such a consequence was predicted for a nearby variant, p.Met184Val, and validated by a severe reduction of MRPS7 protein by western blot [24].

The second variant is predicted to introduce a premature stop codon at p.Lys125. Although this variant is likely to cause nonsense-mediated decay of the corresponding transcript, if the residual transcript persisted and encoded a truncated protein, it would lack the C-terminal portion of the protein, many residues of which are highly conserved (Figure 1d,e and Figures S1–S3).

### 3.3. MRPS7 Variants Are Inherited Bi-Allelically

Given the previously reported patients had bi-allelic variants, indicating a recessive mode of inheritance, we needed to confirm that the two variants identified in our patient were in *trans*. The variants were spaced too far apart to determine this using the available WES data, and parental DNA was not available to confirm compound heterozygosity. We therefore used a cloning strategy to demonstrate that these variants are indeed located on different chromosomes (Figure 1b,c). This is consistent with the patient having bi-allelic variants and *MRPS7* being an autosomal recessive Perrault syndrome/syndromic POI gene.

## 4. Discussion

Here, we describe a patient diagnosed at age 25 with POI after presenting with secondary amenorrhea. She had normal pubertal development, and her ovaries were visible by ultrasound, albeit small. She also had a history of sensorineural hearing loss, diagnosed at age 9. Together, these clinical features are indicative of Perrault syndrome. 

Whole exome sequencing of the patient revealed two heterozygous variants in *MRPS7*, a gene encoding a key component of the 28S small subunit of the mitoribosome. We previously reported an auditory and reproductive phenotype in sisters with a rare homozygous missense variant in *MRPS7*, c.550A>G ( p.(Met184Val)), Table 4 [24], although these patients had a more severe disease course. The correlation between sensorineural hearing loss and hypogonadism observed in these unrelated patients with *MRPS7* variants supports causality.

In addition to hearing loss and POI, both previously reported sisters had other severe symptoms. Patient 1 (P1) presented with lactic acidemia and progressive hepatic and renal failure resulting in death at 14 years and 5 months after the withdrawal of intensive support. Patient 2 (P2) initially had a milder renal and hepatic phenotype. Clinical follow-up of this patient revealed that she experienced progressive decline of liver and renal function with secondary encephalopathy, culminating in a liver and renal transplant at age 25 with a positive outcome. The premature death of P1 prevented insights into her reproductive phenotype, but P2 presented with failed pubertal development and primary hypogonadism. Her TSH was mildly elevated, although she presented clinically euthyroid (Table 2).

The previously reported missense variant and that identified in the current case affect residues only five amino acids apart, c.536G>A; p.(Arg179His) and c.550A>G; p.(Met184Val), and both fall within the predicted Ribosome protein S7 functional domain of MRPS7 which spans codons 82–234 [28]. The Ribosome protein S7 functional domain is responsible for interaction with 12S rRNA in the 3’ head domain of the 28SSU [29]. Unstable small mitochondrial ribosomal subunits prevent 12S rRNA from integrating with 28SSU [24]. As a result, the 12S rRNA is degraded. The previously reported variant in the Ribosome protein S7 functional domain was associated with a significant reduction of 12S rRNA [24]. This suggests that this region of MRPS7 is likely critical for its interaction with 12S rRNA and subsequent mitochondrial protein synthesis. Indeed, variants within 12S rRNA are known to cause non-syndromic deafness [30]. The finding of an additional variant within this functional domain supports its pivotal role in protein function. 

Mitochondrial dysfunction has been observed in many monogenic and polygenic diseases, including cardiovascular, metabolic, neurodegenerative and neuromuscular disorders [31]. The primary role of the mitochondria is to generate energy through oxidative pathways involving fatty acid, glucose and amino acid metabolites via the Krebs cycle and OXPHOS [31]. This process is essential to the generation of ATP, which is critical for cellular function. Therefore, mitochondrial dysfunction has the potential to impact many biological processes but more notably, those processes with high energy demands.

Mammalian ovaries and their constituent oocytes have a particularly high energy demand. Consistent with this, mammalian oocytes have an enriched mitochondrial DNA (mtDNA) copy number when compared to other somatic cells [32]. Oocyte viability has been linked to mitochondrial function, given the role of mitochondria in ATP production, metabolite supply and cell death regulation [33]. Furthermore, pathogenic changes to mitochondria lead to oxidative stress (OS) and elevated reactive oxygen species (ROS) [34]. The accumulation of ROS can unfavourably oxidise RNA, protein and DNA leading to oocyte damage and depletion. OS induces various pro-apoptotic pathways within the oocyte, ovarian follicle, and necroptosis in surrounding granulosa cells [35]. Ovarian follicle atresia leads to rapid depletion of available germ cells within the ovary, resulting in amenorrhea and reproductive cessation. [35].

Given the role of mitochondria in supporting ovarian function, it is not surprising that variants within mtDNA and genes encoding factors involved in mitochondrial protein synthesis have been implicated in ovarian dysgenesis, aberrant folliculogenesis and the depletion of viable oocytes [36]. The case described here consolidates *MRPS7* as a new mitochondrial cause of syndromic POI, supporting the importance of mitochondrial function for ovarian activity.

Ovarian dysfunction is often an under-reported or overlooked symptom of mitochondrial disorder presentation. This is due in part to publication bias, which lead to initial reports describing the most severe cases. Severe mitochondrial disorders often lead to pre-pubertal death. Consequently, reproductive function remains unknown. Given that infertility is not a life-threatening phenotype, it may go unreported in the presence of severe syndromic disease. With the widespread use of WES and rapidly expanding knowledge regarding the full spectrum of genetic diseases, POI is emerging as a recurrent issue for adult patients with milder mitochondrial diseases. In some cases, disruption to mitochondrial function can present as an isolated POI (e.g. MRPS22 deficiency) [23]. The potential for POI as a feature of mitochondrial disorders needs increased awareness because early interventions, such as hormone replacement therapy or oocyte collection, have substantial benefits in preserving fertility and/or long-term health. Establishing *MRPS7* as a syndromic POI gene emphasises the important role of mitochondria for optimal ovarian function. Efficient mitochondrial protein translation appears key to supporting ovarian function, with twelve such genes now being associated with POI pathology [36] (Table 5).

**Figure 2:**
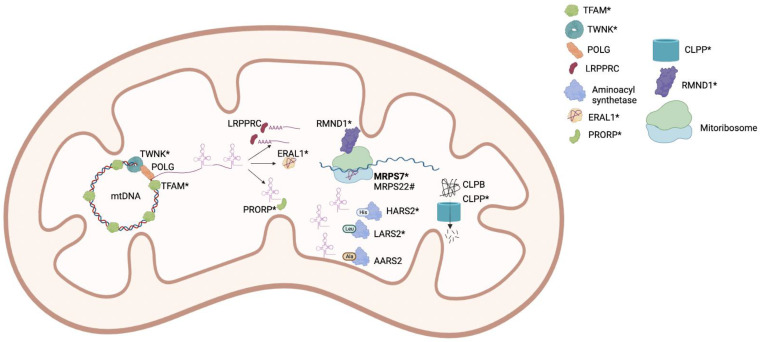
Mitochondrial proteins implicated in Premature Ovarian Insufficiency (POI). Many mitochondrial POI proteins have been associated with Perrault syndrome (indicated with *) with or without additional organ involvement. Bold indicates the MRPS7 protein presented in this study. MRPS7 is an integral component of the small subunit of the mitochondrial ribosome. # indicates MRPS22 which is the only mitochondrial protein associated with isolated POI to date.

**Figure 3:**
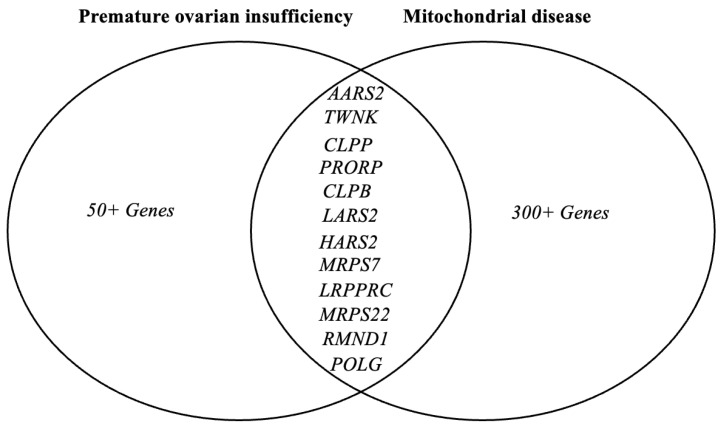
Mitochondria-associated genes in Premature Ovarian Insufficiency (POI). Diagram depicts the intersection of causative mitochondrial genes associated with POI genes, highlighting the contribution of mitochondrial dysfunction in POI [1,52].

The genetic basis of POI as well as Perrault syndrome is incompletely understood. In the case of POI, a genetic diagnosis is only achieved in ~25% of cases. Many POI causative genes are associated with mitochondrial maintenance, translation and/or function (Figure 2. We can now include *MRPS7* as a known mitochondrial cause of POI, expanding the number of causative mitochondrial genes to twelve. Figure 3 depicts the intersection of causative mitochondrial genes associated with POI genes, highlighting the contribution of mitochondrial dysfunction in POI [1,52].

## Figures and Tables

**Table 2:** Clinical summary of proband and updated phenotype of previously reported patient [24].

Patient	Age at Diagnosis	Karyotype	Amenorrhea	Secondary Sex Characteristics	Ultrasound	Auditory Phenotype	FSH (IU/L)	LH (IU/L)	Estradiol (pg/mL)	AMH (ng/mL)	TSH (mIU/L)	Anti-TPO (IU/mL)	Other
Proband	25	XX	Secondary	Normal puberty	Small ovaries, two microfollicles in right ovary absent in left	Sensorineural hearing loss	102	34	29	0.15	12.89	102	Hashimoto’s disease
Sister	21	XX	NR	NR	NR	Congenital hearing loss	NR	NR	NR	NR	NR	NR	NR
Menezes et al. P1	-	XX	NR	NR	NR	Congenital sensorineural hearing loss	NR	NR	NR	NR	NR	NR	Lactic acidemia, progressive hepatic and renal failure
Menezes et al. P2	16	XX	Primary	Failed puberty	Primary hypogonadism	Congenital sensorineural hearing loss	NR	NR	NR	NR	6.75	NR	Mild learning difficulties Renal failure Liver failure Encephalopathy secondary to liver failure. Liver and renal transplant with positive outcome

NR: Not reported, FSH: follicle stimulating hormone, LH: luteinising hormone, AMH: anti-mullerian hormone, TSH: thyroid stimulating hormone, TPO: thyroid peroxidase.

**Table 3:** Variant filtration showing results for gene-centric and variant-centric analysis. Asterisk indicates the *MRPS7* gene that was the leading candidate gene after variant filtration.

	Median Depth	93	
	% Bases > ×10	98.7	
	Criteria	Number of Genes of Interest	
Gene-centric	Moderate-high priority (POI) data	3	*DNAH5, GAB2, YBX2*
Variant centric	Bi-allelic (all)	11	MAP3K6, MMACHC, NBPF14, MAGI1, ALB, PDCD11, SRPR, SOX21, DMXL2, IFT140, MRPS7*
	High priority (all)	17	MAP3K6, RGPD2, HEG1, GYG1, CPEB2, TTC37, GRIFIN, SRPR, SLC38A6, TTC8, BLOC1S6, GOLGA6B, KCNG4, PRA1, YBX2, ATP8B3, MRPS7*

**Table 4:** In-silico variant pathogenicity prediction for *MRPS7* variants.

Patient	gDNA Variant (GrCH38)	cDNA Variant	Protein Variant	Polyphen	Mutation Taster	CADD	SIFT	Provean	ACMG Classification
Proband	chr17: 75263373	c.373A>T	p.(Lys125*)	NA	Disease causing (score 1.000)	Harmful (score 44)	NA	NA	Likely pathogenic
	chr17: 75265730	c.536G>A	p.(Arg179His)	Probably Damaging (score 0.989)	Disease causing (score 1.000)	Harmful (score 23.3)	Damaging (score 0.041)	Neutral (score −2.35)	Likely pathogenic
Menezes et al. [24]	chr17: 75265744	c.550A>G	p.(Met184Val)	Probably Damaging (score 1.000)	Disease causing (score 1.000)	Harmful (score 25.4)	Damaging (score 0.048	Deleterious (score −3.06)	Pathogenic [24]

NA: not applicable.

**Table 5:** Mitochondrial genes associated with premature ovarian insufficiency.

Gene	Inheritance	Clinical Presentation	Gene Function	Molecular Function in Mitochondria
*AARS2*	AR	Ovarioleukodystrophy [37,38]	Aminoacylates alanyl-tRNA	mRNA translation
*CLPB*	AR	Progressive encephalopathy, intellectual disability, epilepsy, congenital neutropenia, cataracts, POI [39]	Caseinolytic peptidase	Mitochondrial matrix Peptidase Chaperone
*CLPP*	AR	Perrault syndrome [40,41]	Mitochondrial matrix protease	Protein degradation
*HARS2*	AR	Perrault syndrome [42,43]	Histidine tRNA	mRNA translation
*LARS2*	AR	Perrault syndrome [44,45]	Leucine tRNA	mRNA translation
*MRPS7*	AR	Perrault syndrome, hepatic and renal failure [24]	Mitochondrial ribosome subunit	Mitochondrial protein synthesis
*MRPS22*	AR	Isolated POI [23]	Mitochondrial ribosome subunit	mRNA translation
*POLG*	AD/AR	Premature ovarian insufficiency, mitochondrial recessive ataxia [46] Premature ovarian insufficiency, chronic progressive external ophthalmoplegia (CPEO) [47]	DNA polymerase *γ*	mtDNA replication
*TWNK*	AR	Perrault syndrome [48,49]	mtDNA Helicase	mtDNA replication and proofreading
*LRPPRC*	AR	Leigh Syndrome [50]	RNA binding protein	Mitochondrial gene expression
*RMND1*	AR	Perrault syndrome, renal, neural, muscular defects [36,51]	Integral membrane protein	Mitochondrial mRNA translation
*PRORP*	AR	Perrault syndrome, developmental delay [6]	Mitochondrial-tRNA processing	Mitochondrial RNA maturation

AR: autosomal recessive, AD: autosomal dominant, tRNA: transfer RNA, mRNA: messenger RNA.

## Data Availability

Some or all datasets generated during and/or analyzed during the current study are not publicly available but are available from the corresponding author on reasonable request. Described variants are submitted to ClinVar (https://www.ncbi.nlm.nih.gov/clinvar/ accessed on 9 October 2022) Accession: SCV002574697–SCV002574698.

## Supplementary Materials

The following supporting information can be downloaded at: https://www.mdpi.com/article/10.3390/genes13112113/s1, Figure S1: Conservation of residue altered by Patient 1 variant c.373A>T. Figure S2: Conservation of residue altered by Patient 1 variant c.536G>A. Figure S3: Conservation of residue altered by Menzes et al patient variant; Table S1: Curation of the variant in Patient 1 using ACMG-based criteria as adopted by the Victorian Clinical Genetics Service (VCGS).
