# Supplementary material for: Integral Role of the Mitochondrial Ribosome in Supporting Ovarian Function: MRPS7 Variants in Syndromic Premature Ovarian Insufficiency
Source: Genes (Basel). 2022 Nov 14;13(11):2113. doi: 10.3390/genes13112113 (PMC9690861; doi:10.3390/genes13112113)
Supplement: Supplementary file 1 [file genes-13-02113-s001.zip › Sup_MRPS7_111022.pdf]

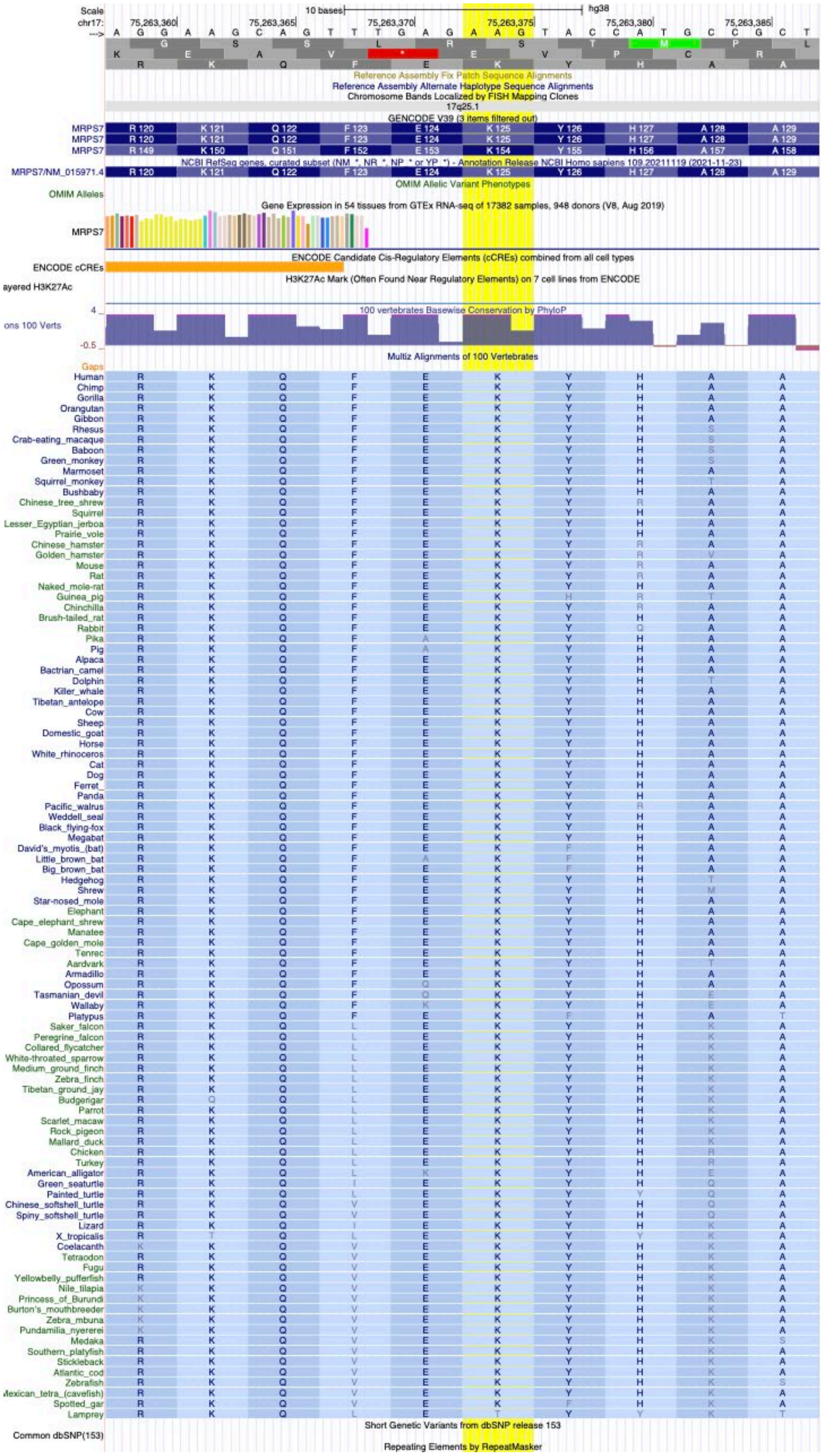

Figure S1. Conservation of residue altered by Patient 1 variant c.373A>T. Multiz Alignment of 100 vertebrates from UCSC Genome Browser https://genome.ucsc.edu/ showing conservation of the p. Lys125 residue altered by the NM_015971.4(MRPS7):c.373A>T, p.(Lys125*) variant in Patient 1.

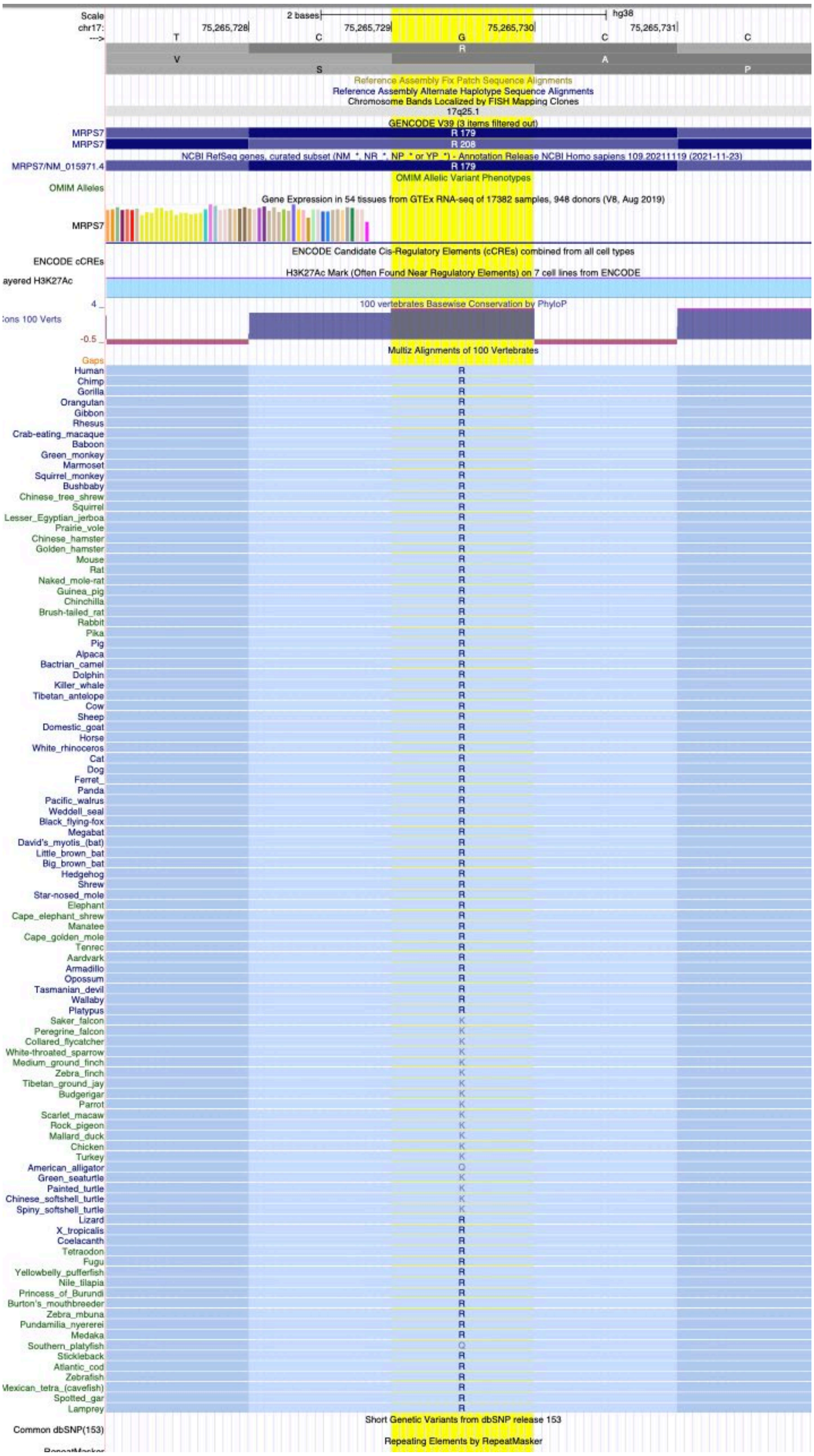

Figure S2. Conservation of residue altered by Patient 1 variant c.536G>A. Multiz Alignment of 100 vertebrates from UCSC Genome Browser https://genome.ucsc.edu/ showing conservation of the p.Arg179 residue altered by the NM_015971.4(MRPS7): c.536G>A, p.(Arg179His) variant in Patient 1.

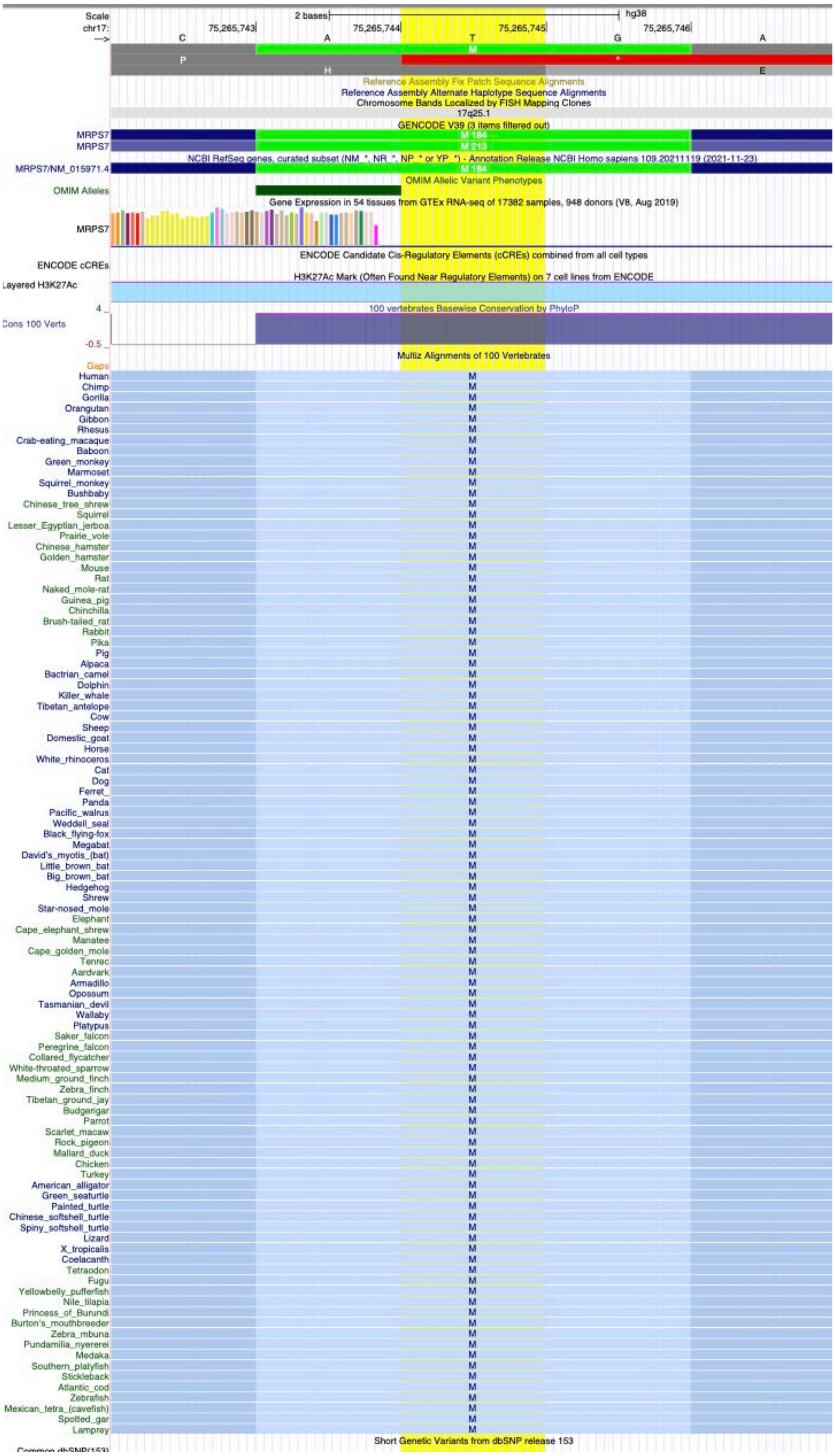

Figure S3. Conservation of residue altered by Menzes et al patient variant. Multiz Alignment of 100 vertebrates from UCSC Genome Browser https://genome.ucsc.edu/ showing conservation of the p. Met184 residue altered by the NM_015971.4(MRPS7): c.550A>G, p.(Met184Val) variant in Menzes et al patient [24].

Supplementary Table S1. Curation of the variant in Patient 1 using ACMG-based criteria as adopted by the Victorian Clinical Genetics Service (VCGS)

**NM_015971.4(MRPS7):c.373A>T, p.(Lys125*)**	
1.1.1: Pathogenic very strong (PVS)	Novel Stop codon is not in the last exon or the last 50bp of the second to last exon
1.7.1: Pathogenic moderate (PM)	Heterozygous variant detected in trans with a second at least likely pathogenic, heterozygous variant in a recessive disease
1.13.1: Pathogenic supporting (PP)	Variant in a gene that is known to be causative for a syndrome with clinical presentation and inheritance pattern consistent with suspected disease. STRONG phenotype match.
Conclusion	1 PVS, 1 PM, 1 PP = Likely pathogenic

**NM_015971.4(MRPS7): c.536G>A, p. (Arg179His)**	
2.1.3: Pathogenic moderate (PM)	MAF for recessive indication <0.01 (use caution with minority ethnicities)
2.3.1: Pathogenic supporting (PP)	In-silico consistently supports pathogenicity (SIFT, PolyPhen2, Mutation Taster, CADD)
2.6.1: Pathogenic moderate (PM)	Heterozygous variant detected in trans with a second at least likely pathogenic, heterozygous variant in a recessive disease
2.13.1: Pathogenic supporting (PP)	Variant in a gene that is known to be causative for a syndrome with clinical presentation and inheritance pattern consistent with suspected disease. STRONG phenotype match.
Conclusion	2 PM + 2 PP = Likely pathogenic
